# Forensic efficiencies of individual identification, kinship testing and ancestral inference in three Yunnan groups based on a self-developed multiple DIP panel

**DOI:** 10.3389/fgene.2022.1057231

**Published:** 2023-01-04

**Authors:** Man Chen, Qiong Lan, Shengjie Nie, Liping Hu, Yating Fang, Wei Cui, Xiaole Bai, Liu Liu, Bofeng Zhu

**Affiliations:** ^1^ Guangzhou Key Laboratory of Forensic Multi-Omics for Precision Identification, School of Forensic Medicine, Southern Medical University, Guangzhou, China; ^2^ Microbiome Medicine Center, Department of Laboratory Medicine, Zhujiang Hospital, Southern Medical University, Guangzhou, China; ^3^ School of Forensic Medicine, Kunming Medical University, Kunming, China; ^4^ School of Basic Medical Sciences, Anhui Medical University, Hefei, China; ^5^ Key Laboratory of Shaanxi Province for Craniofacial Precision Medicine Research, College of Stomatology, Xi’an Jiaotong University, Xi’an, China

**Keywords:** deletion/insertion polymorphism, individual identification, full-sibling identification, extreme gradient boosting, support vector machine

## Abstract

Deletion/insertion polymorphism (DIP), as a short insertion/deletion sequence polymorphic genetic marker, has attracted the attention of forensic genetic scientist due to its lack of stutter, short amplicon and abundant ancestral information. In this study, based on a self-developed 43 autosomal deletion/insertion polymorphism (A-DIP) loci panel which could meet the forensic application purposes of individual identification, kinship testing and ancestral inference to some extent, we evaluated the forensic efficiencies of the above three forensic objectives in Chinese Yi, Hani and Miao groups of Yunnan province. The cumulative match probability (CPM) and combined probability of exclusion (CPE) of these three groups were 1.11433E-18, 8.24299E-19, 4.21721E-18; 0.999610217, 0.999629285 and 0.999582084, respectively. Average 96.65% full sibling pairs could be identified from unrelated individual pairs (as likelihood ratios > 1) using this DIP panel, whereas the average false positive rate was 3.69% in three target Yunnan groups. With the biogeographical ancestor prediction models constructed by extreme gradient boosting (XGBoost) and support vector machine (SVM) algorithms, 0.8239 (95% CI 0.7984, 0.8474) of the unrelated individuals could be correctly divided according to the continental origins based on the 43 A-DIPs which were large frequency distribution differentiations among different continental populations. The present results of principal component analysis (PCA), multidimensional scaling (MDS), neighbor joining (NJ) and maximum likelihood (ML) phylogenetic trees and STRUCTURE analyses indicated that these three Yunnan groups had relatively close genetic distances with East Asian populations.

## Introduction

Deletion/insertion polymorphism (DIP) is an important length polymorphic marker in forensic genetics, it is widespread locus in the whole human genome (Abecasis et al., 2010). Compared with short tandem repeat (STR) (Romanini et al., 2015), DIP is relatively lower mutation rate, no stutter and shorter amplicon, which is suitable to be genotyped using by capillary electrophoresis (CE) platform and increases the possibility to gain genotype information from compromised sample (Tie et al., 2022). In order to enhance the applicability of the detection system for degraded biological sample from crime scene, forensic researchers always try to design the amplicons of molecular genetic markers with less than 200bp (Chen et al., 2019; Jin R. et al., 2021a; Fan et al., 2021). So far, a series of DIP multiplex amplification systems have been developed to meet different forensic objectives like individual identification and kinship testing over the past decade. The Investigator^®^ DIPplex kit was the first commercial DIPplex panel for forensic individual identification, but the 30 A-DIPs included in this kit were low polymorphic in Chinese different groups in several forensic genetic studies (He et al., 2019; Jian et al., 2019; Zou et al., 2020; Jin X. et al., 2021b). Subsequently, several in-house panels including 35 A-DIPs (Jin et al., 2019), and 43 A-DIPs in this study (Jin R. et al., 2021a), and commercial kits including 32 X-DIPs (Gomes et al., 2020), 38 X-DIPs (Chen et al., 2021), AGCU DIP 50 kit (Chen et al., 2019), and AGCU DIP 60 kit (Fan et al., 2021) were developed. A new DIP detection system including 39 ancestral informative DIPs exploited by ourselves was also used to infer the biogeographic ancestry (Jin et al., 2020; Zhang et al., 2021).

The combined probability of exclusion (CPE), cumulative match probability (CPM) and total discrimination power (TDP) are three regular parameters to evaluate the efficiencies for individual identification and paternity testing. Both the likelihood ratio (LR) and identity by state (IBS) methods could be used to calculate the values for kinship testing with biallelic markers such as DIP and single nucleotide polymorphism (SNP), and the method of LR has been proven to be more accurate (Li et al., 2019; Tao et al., 2022). When analyzing the population genetics and ancestral component, STRUCTURE, cluster analysis and principal component analysis (PCA) are mainly used methods (Yang et al., 2019). Machine learning algorithms can be used to solve the problem of prediction and classification of target objects, and show relatively well prediction performances for forensic age prediction (Vidaki et al., 2017), body fluid identification (Liu et al., 2021) and ancestor information prediction (Sun et al., 2022). Among them, supervised learning is a very important algorithm type of machine learning, mainly included linear regression, logical regression, support vector machine (SVM), decision tree, random forest (RF) and extreme gradient boosting (XGBoost) et al.

Yunnan, located in southwest China, is a multi-ethnic province, in which the Yi, Hani and Miao ethnic groups are three numerous minorities. There are more than 5.0 million Yi, 1.6 million Hani and 1.2 million Miao individuals, and these three groups account for 17.11% of the total population of Yunnan province according to the 2010 sixth population census of China. In addition to China, Yi and Hani individuals are also distributed in Vietnam, Burma and other Southeast Asia countries (Jin R. et al., 2021a). According to historical records, after the Ming and Qing Dynasties (650–100 years ago), Miao group settled in Yunnan and Southeast Asia (like Southern Vietnam) and had genetic exchanges with local groups (Wen et al., 2005). In the previous study, our laboratory constructed a novel panel including 43 A-DIP loci and the sex determination locus Amelogenin for individual identification and kinship testing, which showed high forensic efficiencies in Chinese Han and Hui ethnic groups (Jin R. et al., 2021a; Zhao et al., 2022). We performed the present study to evaluate the forensic effectiveness of this panel in three Yunnan (Yi, Hani and Miao) ethnic groups; gain an insight into the genetic backgrounds of three groups; and analyze the genetic affinities among three groups and other reference populations. Fingertip blood samples of 542 unrelated individuals originated from three groups of Chinese Yunnan province were genotyped with the 43 A-DIP panel. Furthermore, we also collected the 43 A-DIP genotype data of 3348 individuals from 28 reference populations from five continents to analyze ancestral components and genetic affinities among the studied three Yunnan groups and those reference populations.

## Materials and methods

### Sample collections

All 542 fingertip blood samples including 125 Yi, 208 Hani and 209 Miao ethnic individuals were collected from Chinese Yunnan province. All the samples were collected with written informed consents. All volunteers who provided blood samples were informed of the purpose and main content of this research; possible risk and many personal rights protecting matters of the sample collection. This research involving human samples was approved by the ethnic committees of Xi’an Jiaotong University Health Science Center, and the approval number is 2019–1039.

### Multiplex amplification and genotyping of the 44 loci panel

A self-developed panel including 43 A-DIP loci and a sex determination locus Amelogenin was used to investigate the genetic diversities of three groups living in Chinese Yunnan province. These 44 loci were evenly designed into four channels labeled with four different dyes, and then 11, 9, 11, 13 loci were labeled by 6-FAM, HEX, TAMRA and ROX, respectively. Additionally, Org 500 (Microread Genetics, Beijing, China) was used to label the internal size standard fragments. The amplification for 44 loci was performed on the 9700 Thermal Cycler (ThermoFisher Scientific, South San Francisco, CA, United States) according to the recommendation in previous work (Jin R. et al., 2021a). The amplicons were detected and separated by the ABI 3500xL Genetic Analyzer and the 3500xL Dx Data Collection Software 3 (ThermoFisher Scientific). All the 44 loci genotypes were generated with the GeneMapper™ ID-X Software v1.5 (ThermoFisher Scientific). To ensure the reliability of the genotyping results, 9947A, 9948 and nuclease-free water were used as positive and negative controls throughout the genotyping process, respectively.

### Data analysis

The Hardy-Weinberg equilibrium (HWE) test and pairwise linkage disequilibrium (LD) analysis were conducted using the Arlequin software v3.5 in three Yunnan groups (Excoffier and Lischer, 2010). Allele frequencies of 43 A-DIPs in three Yunnan groups were calculated with the online tool STR Analysis for Forensic (STRAF) (Gouy and Zieger, 2017). The forensic parameters including polymorphism information content (PIC), matching probability (PM), power of discrimination (PD), expected heterozygosity (He), observed heterozygosity (Ho), power of exclusion (PE) and typical paternity index (TPI) were also calculated using with STRAF. The same 43 A-DIPs genotype data of 28 reference populations were collected to investigate the genetic backgrounds of three studied Yunnan groups, and analyzed genetic relationships among these three groups and other reference populations. The 26 reference populations were collected from the 1000 Genomes Phase III release and other two reference groups from China were collected from the two previous studies (Jin R. et al., 2021a; Zhao et al., 2022). The abbreviations, locations, linguistic families, sample sizes and continents of 28 reference populations and three studied Yunnan groups were shown in Supplementary Table S1. To verify the forensic efficiencies of individual identification and kinship testing, the software familias 3 was used to simulate 1000 full sibling pairs, 1000 half sibling pairs and 1000 unrelated individual pairs based on the allelic frequencies of each ethnic group in this study (Kling et al., 2014). And the familias 3 was also used to calculate the LRs in different relationships. The prosecution hypothesis (H1) is that the two individuals is full sibling pair or half sibling pair, whereas the defense hypothesis (H2) is that the two individuals is unrelated individual pair. And the LR is calculated as the values of H1 divide by H2. Software *R* (https://www.r-project.org/) was used to draw the LR distribution maps of two relationships mentioned above. The cumulative match probability (CPM) is calculated as the formula of CPM = PM_1_ ✕ PM_2_ ✕ PM_3_ ✕ … ✕ PM_k_ (k indicates the total number of loci and the PM_k_ indicates the PM value of the locus k). The total discrimination power (TDP) value is calculated as the formula of TDP = 1 – (1-DP_1_) ✕ (1-DP_2_) ✕ (1-DP_3_) ✕…✕ (1-DP_k_) (k indicates the total number of loci and the DP_k_ indicates the DP value of the locus k). The combined probability of exclusion (CPE) value is calculated as the formula of CPE = 1 – (1-PE_1_) ✕ (1-PE_2_) ✕ (1-PE_3_) ✕…✕ (1-PE_k_) (k indicates the total number of loci and the PE_k_ indicates the PE value of the locus k).

To evaluate the amount of information that these 43 A-DIPs provide about ancestry, the informativeness for assignment (*I*_*n*_) value of population pairwise comparison was conducted among 31 populations with INFOCALC v1.1 (Rosenberg et al., 2003). Four artificial intelligence (AI) algorithms (RF, XGBoost, decision tree, and SVM) were used to build the biogeographic ancestor prediction models. The genotypes of 43 A-DIPs of 3389 individuals were divided into three categories (Africa, East Asia and other). In each category, 75% of the random individuals were used as training set to build model, and the remaining 25% random individuals were test set. The four AI methods were performed on *R* language. Pairwise *F*_*ST*_ distances for short diversity time among three Yunnan groups and other 28 reference populations were generated with the Genepop v4 (Rousset, 2008). The pairwise *Nei’s* genetic distance was calculated with PHYLIP v3.698 https://evolution.gs.washington.edu/phylip.html. The individual-level principal component analysis (PCA) was conducted based on the raw individuals’ genotypes by *R* software. The multidimensional scaling (MDS) analyses were performed by *R* software based on the *Nei’s* genetic distance and pairwise *F*_*ST*_ value, respectively. The neighbor joining (NJ) phylogenetic tree was performed with Mega 10 software based on the pairwise *F*_*ST*_ values (Stecher et al., 2020) and visualized with the inline tool iTOL https://itol.embl.de. The maximum likelihood (ML) phylogenetic tree was conducted among three Yunnan groups and the reference populations using with the TreeMix v1.1 (Pickrell and Pritchard, 2012). And the root of the ML tree was set as Ibadan Yoruba (YRI) which presented the largest pairwise *F*_*ST*_ values with three Yunnan groups. The STRUCTURE v2.3.4 based on Bayesian statistical method was used to evaluate the structure of the three target Yunnan groups and other reference populations based on the 43 A-DIP genotypes with burn-in 10,000 times by 10,000 MCMC repetitions. The predefined numbers of ancestral populations were set from 2 to 5, and each was set as 10-fold cross-validation. The online *R* package tool pophelper (http://pophelper.com) was used to visualize the STRCTURE results based on the individual and population levels.

## Results

In this study, 542 unrelated individuals including125 Yis, 208 Hanis and 209 Miaos from three Yunnan groups were genotyped with the 43 A-DIPs panel. The Hardy-Weinberg equilibrium and linkage disequilibrium tests, DNA polymorphism, PD and PM of each locus, CPD and CPM were all calculated to assess forensic efficacy of individual identification of the 43 A-DIP panel in three Yunnan groups. Additionally, although this panel was designed for individual identification, some loci also represented the potential to infer ancestry information. In order to fully exploit the forensic performance of the self-developed 43 A-DIP panel in three target groups, the ancestry components and population genetic relationships among three Yunnan groups and 28 reference populations were analyzed.

### Hardy-Weinberg equilibrium and linkage disequilibrium tests of all DIP loci in three Yunnan groups

The HWE statuses of the 43 A-DIP loci were used to assess the suitabilities of the 43 loci in Yunnan Yi, Hani and Miao groups. And the *p* values and standard deviation (SD) results of Hardy-Weinberg equilibrium tests in three Yunnan groups were shown in Supplementary Table S2. No significant deviations from Hardy-Weinberg equilibrium were identified at 43 A-DIP loci in these three groups after the Bonferroni correction (0.05/43 = 0.0012). The LD tests were conducted among 43 A-DIP combinations, and the pairwise *p* values of LD tests at 43 A-DIPs in the Yi, Hani and Miao groups were all shown in Supplementary Tables S3–S5, respectively. After the Bonferroni correction (*p* < 0.0001 = (0.05*2)/(43*42)), all the pairwise loci of 43 A-DIPs were in accordance with the linkage equilibrium.

### Allelic frequencies and forensic parameters of 43 A-DIPs in Yunnan three groups

**FIGURE 1:**
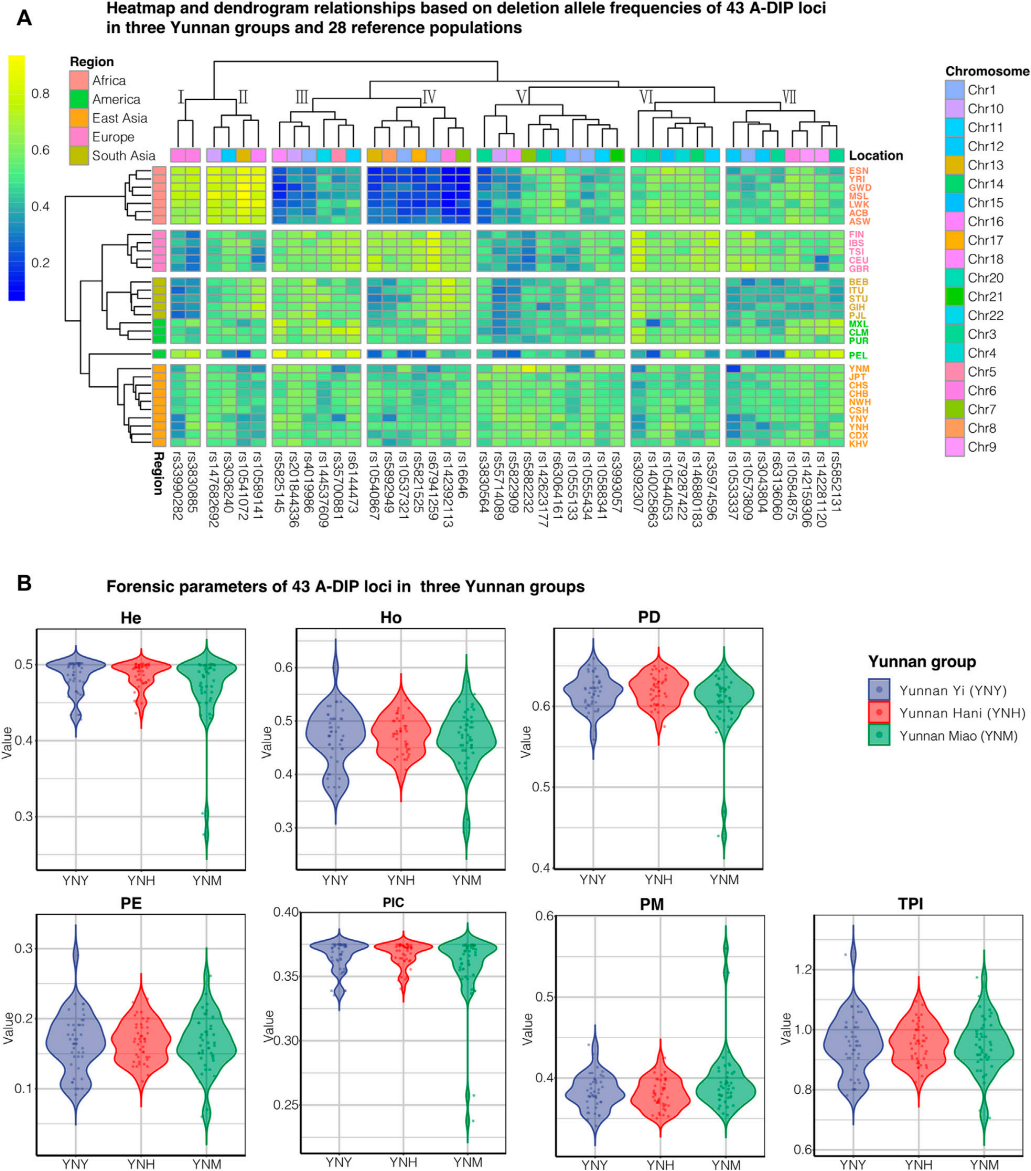
The deletion allelic frequency heatmap and forensic parameters of 43 A-DIPs in three Yunnan groups. **(A)** The deletion allelic frequency heatmap of 43A-DIP loci in Chinese Yunnan Yi, Hani, Miao groups and 28 reference populations; **(B)** The forensic parameters including He, Ho, PD, PE, PIC, PM and TPI of Chinese Yunnan Yi (YNY), Hani (YNH), and Miao (YNM) groups with blue, red, and green, respectively.

The deletion and insertion allelic frequencies of the 43 A-DIP loci in the Yunnan Yi, Hani and Miao groups were shown in Supplementary Table S6. In Yi group, the average deletion allelic frequency was 0.4993 (SD = 0.0954), and the deletion frequencies ranged from 0.3080 at rs33990282 to 0.6480 at rs63064161; in Hani group, the average deletion allelic frequency was 0.5141 (SD = 0.0874), and the smallest deletion frequency was 0.3197 at rs33990282 while the largest deletion frequency was 0.6010 at rs10544053 locus; in Miao group, the average deletion allelic frequency was 0.5092 (SD = 0.1261), and the deletion allelic frequencies ranged from 0.1866 (rs10533337) to 0.8349 (rs5882232). The heatmap of deletion allelic frequencies of Chinese three Yunnan groups and 28 reference populations was shown in Figure 1A. Populations from different continents could be identified by different colored fonts on the right and different colored blocks on the left. It could be seen from the clustering results on the left, 30 populations could be clustered into four clusters, and populations from the same continent gathered together and separated from others, except for the American mixed populations.

The forensic parameters of 43 A-DIP loci including PIC, PM, PD, He, Ho, PE and TPI of Chinese Yunnan Yi, Hani and Miao groups were calculated and presented in Figure 1B and Supplementary Table S7, respectively. The PD values varied from 0.4397 (rs5882232, YNM) to 0.6592 (rs10584875, YNY); the PM ranged from 0.3408 (rs10585875, YNY) to 0.5603 (rs5882232, YNM); and the PE varied from 0.0603 (rs5882232, YNM) to 0.2909 (rs35974596, YNY), respectively. The maximum PIC value was 0.3750 (rs5892949, YNH), while the minimum value was 0.2377 (rs5882232, YNM). The He and Ho values were in the range from 0.2763 (rs5882232, YNM) to 0.5020 (rs3830564 and rs147682692, YNY), 0.2919 (rs5882232, YNM) to 0.6000 (rs35974596, YNY). The TPI values ranged from 0.7061 (rs5882232, YNM) to 1.2500 (rs35974596, YNY).

### The forensic efficiencies of individual identification and kinship testing of 43 A-DIP panel

To verify the forensic efficiencies of this panel for the individual identification in three target Yunnan groups and 28 reference populations, the CPM, TDP and CPE were calculated and their values were shown in Supplementary Table S8. The CPM values of Yi, Hani and Miao groups were 1.11433 E^−18^, 8.24299 E^−19^ and 4.21721 E^−18^; the TDP and CPE values of the corresponding groups were 0.99999999999999998886, 0.999999999999999991757 and 0.99999999999999995783; 0.999610217, 0.999629285 and 0.999582084, respectively. Considering all the 3890 individuals from five continents as a whole, the CPM, TDP and CPE values of the 43 A-DIP panel were 1.74469 E^−19^, 0.999999999999999998255, 0.999585879, respectively. Among the continents, the East Asian populations represented the largest average CPE (0.999820028167545) and TDP values (0.99999999999999995194), and the smallest CPM value (2.37739474441527E-18). Whereas, the smallest CPE (0.998911970139209) and TDP (0.9999999999999931467), and the largest CPM (2.87186303945698E-16) were observed, indicating relative lower forensic individual identification efficiency in African populations.

**FIGURE 2:**
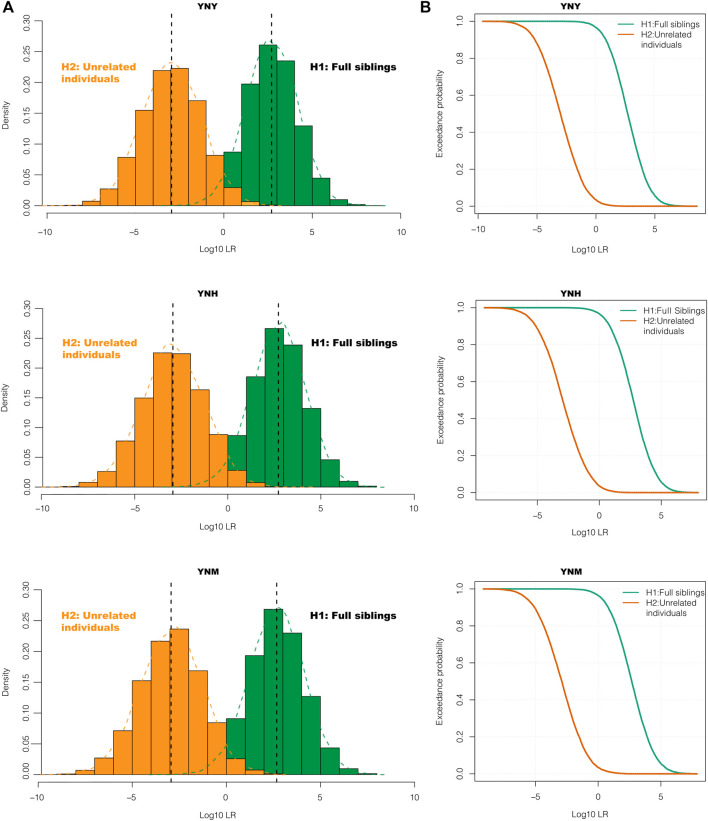
The probability density curves **(A)** and log_10_LR density distribution curves **(B)** of full-sibling pairs and unrelated individual pairs with the 43 A-DIPs. The LR values of prosecutive hypothesis H1 and defense hypothesis H2 for Yunnan Yi (YNY), Yunnan Hani (YNH) and Yunnan Miao (YNM) groups were shown from the top to the bottom.

The effectiveness of kinship testing was also an important index of the individual identification panel. The system efficacies of 43 A-DIP panel for full sibling and half sibling relationships were assessed. Genotypes of 1000 full sibling pairs, 1000 half sibling pairs and 1000 unrelated individual pairs based on 43 A-DIP loci were simulated and used to calculate the LRs of prosecutive hypothesis H1 and defense hypothesis H2. The LR was designed to be the prosecutive hypothesis H1 (full sibling pairs, half sibling pairs) divided by the defense hypothesis H2 (unrelated individual pairs). The distribution curves and probability density distributions of LRs between full sibling pairs and unrelated pairs in these three groups were shown in Figure 2. The LR distribution curves and probability density distributions of full sibling pairs were distinct from those of unrelated individual pairs. The accuracy rates and false positive rates of the full sibling and half sibling determinations based on 43 A-DIP loci with the method of LR were shown in Table 1. And the LR limits were set as 1, 10, 100, 1000 and 10000, respectively. Among Yunnan Yi, Hani and Miao groups, there were similar discriminative abilities for full siblings based on the 43 A-DIPs panel. Average 96.65% full sibling pairs could be identified from unrelated individual pairs (LR > 1), while the average false positive rate was 3.69%; When the H1/H2 LR limits were set as 10, 100, 1000 and 10000, the average accuracy rates were 87.94%, 68.20%, 42.12% and 18.62%, whereas the corresponding average false positive rates were 0.76%, 0.10%, 0.00% and 0.00%, respectively. Additionally, the 43 A-DIPs panel could only distinguish half sibling relationship to a certain extent. When the H1/H2 LR limits were set as 1, 10, 100, the average accuracies of half sibling identifications in three Yunnan groups were 80.7%, 20.2% and 1.67%, respectively.

**TABLE 1:** The accuracy rates and false positive rates of the full sibling and half sibling identifications based on 43 A-DIPs frequencies with the method of likelihood ratio.

Relationship	Group	LR limit (1)		LR limit (10)		LR limit (100)		LR limit (1000)		LR limit (10000)	
		AR	FPR	AR	FPR	AR	FPR	AR	FPR	AR	FPR
Full sibling	Yunnan Yi	96.65%	3.69%	87.94%	0.76%	68.20%	0.10%	42.12%	0.00%	18.62%	0.00%
	Yunnan Hani	96.67%	3.62%	88.00%	0.82%	69.46%	0.07%	42.81%	0.01%	18.93%	0.00%
	Yunnan Miao	96.42%	3.48%	87.32%	0.90%	68.00%	0.15%	41.17%	0.00%	18.19%	0.00%
Half sibling	Yunnan Yi	80.10%	19.90%	28.90%	2.00%	2.50%	0.00%	—	—	—	—
	Yunnan Hani	81.50%	20.40%	29.10%	1.50%	1.60%	0.10%	—	—	—	—
	Yunnan Miao	80.50%	20.30%	29.40%	1.50%	2.00%	0.00%	—	—	—	—

“AR”, the accuracy rate; “FPR”, the false positive rate; “—“, the system cannot identify the half sibling relationship under the LR limits.

### The potential for biological ancestry informative inference of 43 A-DIP panel

Even though the 43 A-DIP panel was originally developed for forensic individual identification, it was still found that the allele frequency distributions of several loci were different in several continental populations, which might have the potential to infer ancestry informative.

### Allelic frequency distributions and *I*_*n*_ values in 31 populations

In the clustering heatmap of 43 A-DIP deletion allelic frequencies in three Yunnan groups and 28 reference populations was shown in Figure 1A, it could be seen that populations from the same continent congregated with each other and separated from other intercontinental ones. Additionally, the *I*_*n*_ values among Yunnan Yi, Hani and Miao groups and other reference populations were calculated and shown in Supplementary Tables S9–S11, respectively. The DIP loci in cluster Ⅳ (rs10537321, rs10540867, rs142392113, rs16646, rs5821525, rs5892949, and rs67941259) showed relatively low frequencies in African populations and high frequencies in East Asian populations. And the DIP loci in cluster Ⅳ also represented high *I*_*n*_ values among three Yunnan groups and African populations, which might be adopted as ancestral informative markers to differentiate African populations and non-African ones. As a contrary, rs33990282, rs3830885 loci in cluster Ⅰ and rs10541072 and rs10589141 in cluster ⅠⅠ exhibited high deletion allelic frequencies in African populations and relatively low deletion frequencies in European, Asian, and American populations, indicating which could be used to differentiate the African and non-African populations. Overall, the 43 A-DIP panel was the potential to infer the individual biogeographic ancestry informative and could be also used to analyze the population genetic differentiations and similarities to some extent.

### STRUCTURE analyses of three Yunnan groups and 28 reference populations

**FIGURE 3:**
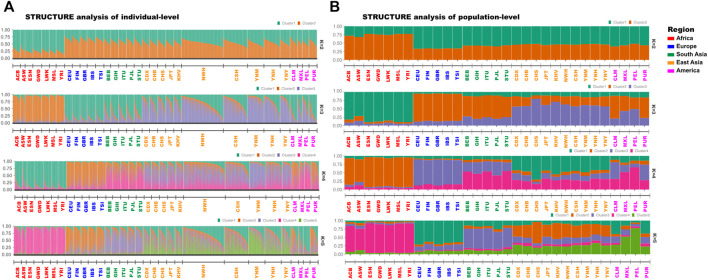
The results of STRUCTURE analyses of Yunnan Yi, Hani and Miao groups and 28 reference populations. **(A)** STRUCTURE results of individual-level were studied based on genotypes of 3890 individuals from five continents. And the individuals among different populations were ordered according to the proportion of cluster one; **(B)** STRUCTURE results of population-level were analyzed based on 43 A-DIP genotype data of 31 populations from five continents.

The STRUCTURE analyses on individual (Figure 3A) and population-levels (Figure 3B) were performed to evaluate the ancestral compositions of Yunnan Yi, Hani and Miao groups. There were no outliers among three Yunnan groups and 28 reference populations in the results of STRUCTURE analysis on individual-level. And then we turned to focus on the STRUCTURE analysis on population level. When *K* = 2, the African ancestral component (orange - cluster 1) and the non - African ancestral component (peacock green - cluster 2) were identified from the African populations and non - African populations. As *K* = 3 (the optimal *K* value), the purple ancestral component (cluster 3) appeared in European, South Asian, East Asian and American populations, and the European populations could be distinguished from others. When the *K* values increased from 4 to 5, the proportions of shared ancestral components varied among different continental populations, but no significant substructures were identified within three target Yunnan groups and other East Asia populations.

### Efficiency of biogeographical ancestor prediction models constructed with four AI algorithms of 43 A-DIP panel

Four AI algorithms including XGBoost, RF, KVM, and decision tree were used to predict the biogeographical ancestors based on the 43 A-DIP loci genotypes of three Yunnan groups and 28 reference populations. The confusion matrixes of the testing sets, the accuracies and 95% confidence interval (95% CI) results of four models were shown in Table 2. Based on the STRUCTURE results of ancestral compositions at the optimal *K* value of 3, we divided all the 3890 individuals from three target groups and 28 reference populations into three categories which included Africa, East Asia and the other continents. The accuracies and 95% CI values of XGBoost, RF, KVM and decision tree methods in judging the origins of three different biogeographic ancestors (Africa, East Asia and others) were 0.8239 (95% CI 0.7984, 0.8474), 0.8126 (95% CI 0.7866, 0.8366), 0.8239 (95% CI 0.7984, 0.8474) and 0.688 (95% CI 0.6578, 0.717), respectively.

**TABLE 2:** The accuracies and 95% CI values of four artificial intelligence (AI) models including the XGBoost (A), RF (B), KVM (C) and decision tree (D) as for the biogeographical ancestor prediction.

A Extreme Gradient Boosting (XGBoost)				B Random Forest (RF)			
Accuracy: 0.8239 95% CI: (0.7984, 0.8474)				Accuracy: 0.8126 95% CI: (0.7866, 0.8366)			
Prediction	Africa	East Asia	Other	Prediction	Africa	East Asia	Other
Africa	152	12	6	Africa	145	4	3
East Asia	9	402	82	East Asia	16	416	103
Other	4	58	246	Other	4	52	228

C Support Vector Machine (SVM)				D Decision Tree			
Accuracy: 0.8239 95% CI: (0.7984, 0.8474)				Accuracy: 0.688 95% CI: (0.6578, 0.717)			
Prediction	Africa	East Asia	Other	Prediction	Africa	East Asia	Other
Africa	148	8	3	Africa	104	15	7
East Asia	15	402	81	East Asia	48	355	118
Other	2	62	250	Other	13	102	209

### Genetic relationships among Yunnan Yi, Hani and Miao groups and 28 reference populations on the basis of 43 A-DIPs

Due to the existence of these loci with large differences in allele distributions, the analyses of PCA, MDA and phylogenetic trees (NJ and ML) were performed to gain insight into the genetic structures and genetic relationships among Yunnan Yi, Hani and Miao groups and other continental populations.

### Genetic affinities among Yunnan Yi, Hani and Miao groups and 28 reference populations

**FIGURE 4:**
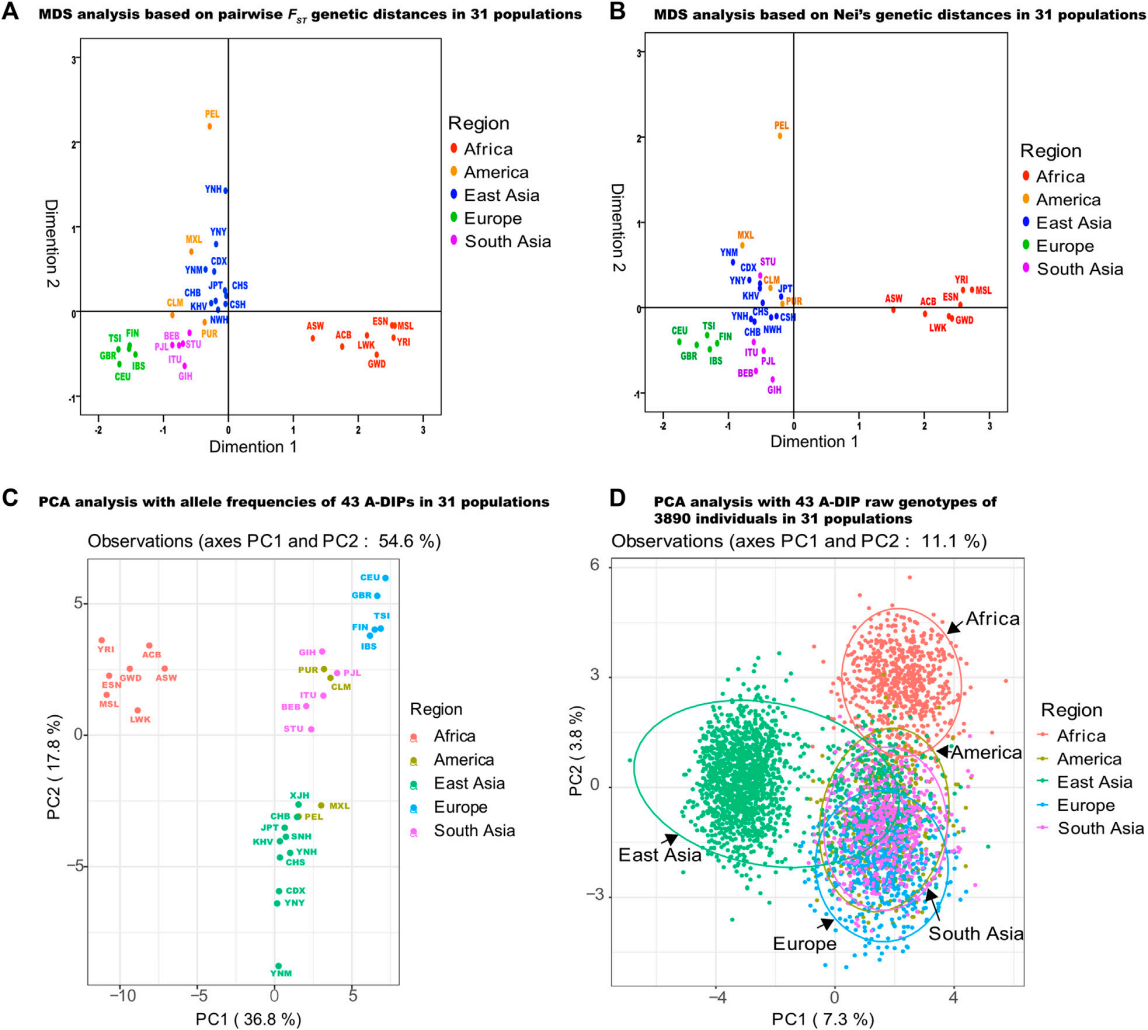
MDS and PCA analyses of three Yunnan groups and 28 reference populations based on 43 A-DIPs. **(A)** MDS analysis based on pairwise *F*_*ST*_ genetic distances among Yunnan Yi, Hani, Miao groups and 28 reference populations; **(B)** MDS analysis based on the *Nei’s* genetic distances among 31 populations; **(C)** PCA analysis based on the allele frequencies of 31 populations; **(D)** PCA analysis based on the 43 A-DIP genotype data of 3890 individuals from five continental populations.

Genetic affinities among Yunnan Yi, Hani and Miao groups and 28 reference populations were evaluated with the pairwise *F*_*ST*_ values (Supplementary Table S12), *Nei’s* genetic distances (Supplementary Table S13), MDS (Figures 4A,B) and PCA (Figures 4C,D) methods. The largest three pairwise *F*_*ST*_ values among Yunnan Yi, Hani groups and 29 reference populations were both observed in YRI, Mende in Sierra Leone (MSL), and Esan in Nigeria (ESN), whereas the smallest three values were found in Xishuangbanna Dai Chinese (CDX), Ho Chi Minh Kinh (KHV), and Chinese Guangzhou Southern Han (CHS). The YRI, MSL and Gambian in ern Division-Mandinka (GWD) were three populations which showed the three largest pairwise *F*_*ST*_ values with Yunnan Miao group. The CDX, CHS and YNY populations had the closest genetic distances with Miao group, and those populations were also observed the smallest three pairwise *F*_*ST*_ values. The *Nei’s* genetic distances among Yunnan Yi, Hani, Miao and 28 reference populations were in ranges from 0.00564 (YNH) to 0.1281 (MSL), 0.0056 (YNY) to 0.1254 (MSL), and 0.0129 (YNY) to 0.1461 (MSL), respectively. MDS analyses based on the matrices of pairwise *F*_*ST*_ values and *Nei’s* genetic distances among 31 populations were shown in Figures 4A,B, respectively. It could be seen that other than the American populations with mixed evolution origin, populations from Africa, Europe, East Asia and South Asia could be clustered together in a certain extent. The PCA analysis based on the allele frequencies of 31 populations was shown in Figure 4C. The first three principal components could explain a total of 67.4% of the differences in allelic frequency distributions among populations, of which the first principal component (PC1) explained 36.8%, the second principal component (PC2) explained 17.8%, and the third principal component (PC3) explained 12.8%. The PCA analysis based on all 3890 individual’s genotypes was shown in Figure 4D, and the first three components could explain 10.4% of the differences among individual’s genotypes, as the PC1, PC2 and PC3 could explain 7.3%, 2% and 1.8%, respectively.

### Phylogenetic trees of Yunnan Yi, Hani and Miao groups and 28 reference populations

**FIGURE 5:**
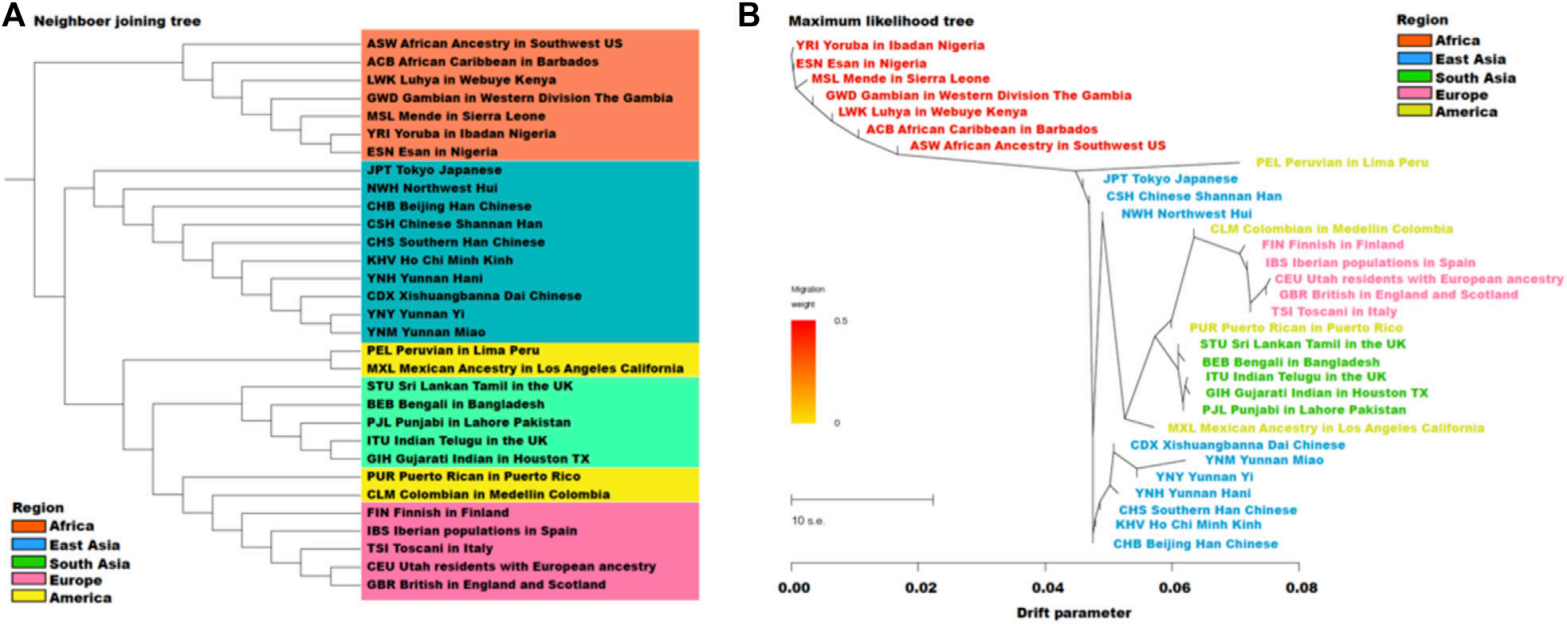
The neighbor joining tree **(A)** based on pairwise *F*_*ST*_ genetic distances, and maximum likelihood tree **(B)** based on allele frequencies among Yunnan Yi, Hani, Miao groups and 28 reference populations.

To evaluate the genetic differentiations and similarities among three target groups and 28 reference populations, we constructed two phylogenetic trees including the NJ tree and the ML tree (Figure 5), which were generated based on the pairwise *F*_*ST*_ genetic distances and allele frequencies among Yunnan Yi, Hani and Miao groups and 28 reference populations, respectively. In the NJ tree in Figure 5A, all 31 populations gathered into three clusters including the African, East Asian and other populations. The Yunnan Yi and Miao groups showed close genetic distance, which gathered with the Chinese Xishuangbannai Dai (CDX), and then clustered with Yunnan Hani group.

Populations which geographically located at the Southern East Asia, like YNY, YNM, CDX, YNH, KHV and CHS gathered together. Except mixed American populations, in ML tree (Figure 5B), from the root of YRI, the African populations showed firstly, then the branch of East Asian populations located at Northern area Tokyo Japanese (JPT), Chinese Shannan Han (CSH) and Chinese Northwest Hui (NWH) was appeared, and then the branches of East Asian located at Southern area, South Asian and European populations were showed, respectively. In the ML tree, ten populations from East Asia were divided into two clusters, the first one consisted of Tokyo Japanese (JPT) and two groups from Northwest China, CSH and NWH; in the second cluster, three Yunnan groups showed the closest genetic distances to CDX, and then combined with other three East Asia populations (CHS, KHV and CHB).

## Discussion

In this study, we genotyped 125 Yi, 208 Hani and 209 Miao individuals from Chinese Yunnan province based on a self-developed 43 A-DIP panel to verify its forensic efficiencies for individual identification and kinship testing. Furthermore, the potential of ancestral information inference with 43 A-DIP panel was also evaluated with four AI models based on the population genetic data of 1000 Genomes Phase III and several previously self-detected reference populations. Additionally, we got insight into the genetic structures of three target groups and explored genetic affinities among three target groups and other reference populations.

None of the 43 A-DIP loci deviated from the HWE in Yunnan Yi, Hani and Miao groups. The LD results of pairwise loci showed that there were non-random associations at 43 different DIP loci. The HWE and LD results indicated all the 43 A-DIP loci were independent each other in the Yunnan Yi, Hani and Miao groups, indicating that the allele frequency data obtained in this study were suitable for the directly LR calculations of individual identification and kinship testing.

The 43 A-DIP panel showed that all the DIPs were relatively high polymorphisms and it could play an effectiveness role in the forensic individual identification, paternity testing and kinship analysis in Yunnan Yi, Hani and Miao groups. In three target Yunnan groups, the average deletion allele frequency of these three groups was 0.5080. In other 28 reference populations, the deletion frequencies of 43 A-DIPs were all close to 0.5, showing relatively high polymorphisms of these biallelic genetic markers. The CMP and CPE values of the 43 A-DIP loci in Yunnan Yi, Hani and Miao groups were 1.11433E-18 and 0.999610217, 8.24299E-19 and 0.999629285, 4.21721E-18 and 0.999582084, respectively, which also indicated that the joint analysis of the 43 A-DIPs was helpful for individual identification and paternity testing. Compared with the 30 DIPs (Yang et al., 2019), 35 DIPs (Cui et al., 2020) and 39 DIPs (Jin et al., 2020) panels, the higher CPE values were obtained by the 43 A-DIP panel which might indicate stronger efficiencies of individual identification, while the ability was comparable to that of 50 DIPs system (Liu et al., 2020; Wang et al., 2021). We applied 43 A-DIPs to perform the full-sibling identification, it could be seen from the LR calculations of the 1000 simulated families, that the 43 A-DIP panel could also provide meaningful conclusion when facing with the full sibling testing case.

Because of the existence of some loci with large differences in allele frequencies among different continental populations, such as rs33990282, rs10541072, rs10541072, 43 A-DIP loci were the potential of ancestral informative inference. With the optimal *K* value was three in the STRUCTURE analyses, the ancestral components of different continental populations could be divided into the African component, East Asian component and mixed component. But there were no substructures observed in three target Yunnan groups and East Asia populations. Four AI models (XGBoost, RF, KVM and decision tree) were used to predict biogeographic ancestors based on the 43 A-DIPs genotypes, the XGBoost and KVM showed the highest accuracies, which were all 0.8239 (95% CI 0.7984, 0.8474). SVM is mainly used to solve the binary classification and regression problems of small samples (Huang et al., 2018). XGboost algorithm is derived from decision tree algorithm and it is a kind of integrated learning algorithm based on gradient boosting, it achieves accurate classification effect through iterative calculation of weak classifier, and at last the prediction result is concluded from all the accumulated conclusion of multiple decision trees (Hatwell et al., 2020). Compared with the algorithms of RF and decision tree, XGBoost and KVM might be more appropriate for ancestral inference based on the multiple DIP genotypes. The model of XGBoost also showed the better prediction performance in the evaluation of biogeographic ancestry inference comparing with the AI models of logistic regression, SVM, and k-Nearest Neighbor based on 15 DIP genotypes generated from the previous study, (Sun et al., 2022).

Population genetic structures and population genetic relationships of three target Yunnan groups and 28 reference populations were also analyzed based on the 43 A-DIP genotypes. The Yi, Hani and Miao groups we collected were all from Yunnan province which are located at the Southwestern China, and were geographically close to Xishuangbanna Dai populations. Three Yunnan groups showed smaller *Nei*’s genetic distances between each other, and indicated closer genetic relationships. From the results of pairwise *F*_*ST*_ genetic distances, other than the close genetic relationships were found among the three studied Yunnan groups, smaller *F*_*ST*_ values were observed among the CHS, CDX and KHV with Yunnan Yi, Hani and Miao groups, which were consistent with the relatively geographic distributions. In the PCA and MDS plots, the Yunnan Yi, Hani and Miao groups had closer genetic distances with other East Asia populations, and among seven East Asian populations, the Southwestern Chinese populations CDX and CHS were the two close ones. Among the NJ and ML phylogenetic trees, Yunnan Yi, Miao and Hani groups represented closer genetic relationships with CDX, CHS and KHV comparing with other reference populations. And similar conclusion was been verified in other study. For instance, Cui et al. reported the NJ and ML trees based on 39 AIM-DIP panel and concluded that the YNM and YNH groups had the close genetic distances with CDX, CHS and KHV (Cui et al., 2022).

## Conclusion

The 43 A-DIP panel showed the relatively high discrimination power of individual identification and played a potential role in the full-sibling testing in Yunnan Yi, Hani and Miao ethnic groups. Additionally, we provided the valuable data of the allele frequency information of 43 relatively high polymorphic DIP loci in three Yunnan groups for further forensic applications. The AI models based on XGBoost and KVM had been verified with higher accuracy for the prediction of biogeographical ancestor based on the 43 DIP genotypes. Through the conjoint analyses of the genotypes of 3348 individuals from 28 reference populations, the Yunnan Yi, Hani, Miao groups had relatively close genetic relationships with KHV, CDX and CHS. In conclusion, the 43 A-DIP panel was a powerful tool for forensic individual identification, full sibling testing and biogeographical ancestral inference in Yunnan Yi, Hani and Miao groups.

## Data Availability

The datasets for this article are not publicly available due to concerns regarding participant/patient anonymity. Requests to access the datasets should be directed to the corresponding author.
